# Use of Maraging Steel 1.2709 for Implementing Parts of Pressure Mold Devices with Conformal Cooling System

**DOI:** 10.3390/ma13235533

**Published:** 2020-12-04

**Authors:** Jarosław Piekło, Aldona Garbacz-Klempka

**Affiliations:** Faculty of Foundry Engineering, AGH University of Science and Technology, Al. A. Mickiewicza 30, 30-059 Kraków, Poland; jarekp60@agh.edu.pl

**Keywords:** die casting, selective laser melting, maraging steel, conformal cooling, heat treatment, mechanical properties, microstructure, FEM calculations

## Abstract

In this paper, we present the results of experimental tests and numerical calculations for parts of foundry mold devices made by selective laser melting (SLM). The main aim of this research was to compare the heat conduction efficiency of the conformal and the traditional channel arrangement. The infusion spreader with a conformal channel arrangement and the test material were made with an M2 Concept Laser Cusing machine using 1.2709 steel powder. Temperature changes in the spreaders were compared between conventional and conformal cooling channels using finite element method (FEM) calculations. The position of the so-called “thermal equilibrium isotherm” was determined for both sprue spreaders, which separate the area of the mold with a constant temperature from the zone of cyclic temperature changes. The components of the sprue spreaders in a stress state caused by temperature changes during the operation of the pressure machine were determined using the FEM model. It was found that the cooling system shortened the time of solidification and cooling of the alloy. Based on the analysis of the strength test results and the fracture surface of the samples, the relationship between heat treatment parameters and the strength, hardness, and elongation of the tested material was determined. The sprue spreaders were installed under a pressure machine and tested under production conditions. The use of a sprue spreader with a conformal cooling system shortened the time of a single cycle of the casting machine compared to the conventional solution.

## 1. Introduction

A pressure mold is a complicated and precise tool that is designed to work directly in contact with liquid metal and used to shape it under high pressure in a mold cavity. An important issue is properly cooling the mold, which absorbs heat from the solidifying alloy. A conventional cooling solution consists of straight-line-channels made with the use of computerized numerical control (CNC) machining. Thanks to conformal channels, the trajectory of which are adjusted to the form of the surface to be cooled, the efficiency of heat transfer from the solidifying alloy is more effective [1,2,3,4,5]. To create a conformal cooling channel system, selective laser melting (SLM) can be used, providing a designer with more freedom in terms of designing the shape, position, and course of cooling ducts [6,7,8,9,10,11,12,13,14]. In plastic injection molds produced by the SLM method, inserts and cores with a conformal cooling system are increasingly being utilized. Here, we aim to examine whether the SLM method and conformal cooling system can also be successfully used in metal casting, where applications of additive technology have been rarely studied. In the cavity of the pressure mold, there are significantly greater pressures and temperatures than in the case of injection molds. Thus, we analyzed both the strength properties of the material obtained by the SLM method, the transport of heat in the mold, the state of thermal stresses, and the results of production tests at the present stage of their implementation. The research focused on the cooling part of the gating system, namely, the sprue spreader, the upper surface of which is in thermal contact with the “biscuit”, which is approximately 30 mm thick. By increasing the intensity of heat transport from the solidifying biscuit, we aimed to reduce the cycle time of the pressure machine. The pressure mold machine consists of many elements that are designed to ensure its functionality and proper operation. Most of the elements included in a mold are made of constructional carbon steels, with some variability in terms of their type depending on the type of cast alloy; however, those elements that come into contact with liquid metal are made from hot work steel. The fixed and ejector die half of the die, as well as the cores and spreaders, are usually made of H13 steel, which was recommended for manufacturing the parts of pressure molds by the North American Die Casting Association (NADCA) [15]. Recently, precipitation-strengthened low-carbon martensite steel of the maraging type with the Marlok C1650 symbol has been used for mold manufacturing. The chemical composition of this steel is similar to the chemical composition of the CL 50WS powders used by the Concept Laser company, as well as the MS1 powders used by the Electro Optical Systems (EOS) company to produce tooling parts that work at high temperatures using the SLM method. The application scope of these powders includes creating the inserts and cores of pressure molds for the casting of aluminum alloys. Table 1 presents a comparison of the chemical composition of the above-mentioned steels and powders.

Maraging steels possess many advantages, including high resistance to cracking, high strength at temperatures of up to 500 °C, ease of machining, and relatively low aging temperatures. Another advantage is the lack of dimensional changes after heat treatment. Apart from its good mechanical properties, it is also characterized by good “weldability”, which is required for the SLM process [20]. The term weldability mainly relates to the low tendency of an alloy to form cracks during solidification and cooling [21,22]. When assessing weldability, the role of the chemical composition of the steel is dominant. According to [23], the low carbon (C) content in maraging steel prevents corrosion and hardening fractures. Harmful additives, such as sulfur (S) and phosphorus (P), impair a material’s weldability; therefore, their content should be kept to a minimum.

The good weldability of maraging steel can be explained by the lack of elements that form interstitial solutions within the alloy [24]. Due to rapid alloy cooling (10^3^–10^6^ °C/s) during the SLM process, the resulting material possesses a martensitic structure, although studies that take advantage of optical microscopy and SEM do not show the presence of a typical form of martensite [25]. This material possesses good machinability due to its relatively low Rockwell C Hardness (HRC), which is between 30 and 40 in its raw state. Heat treatment ages an alloy and improves its strength and hardness as a result of separating the intermetallic phases [26]. The Ni_3_Ti, Ni_3_Mo, and Fe_7_Mo_6_ intermetallic phases, which all have a fundamental effect on the strengthening of the alloy, are formed at an aging temperature in the range of 400–490 °C [27,28,29,30].

There are many phenomena that restrict die life, the most important being thermal fatigue, corrosion, erosion, cracking, and indentation. Thermal fatigue cracks are caused by a combination of thermal cyclic stresses, mechanical stresses, and plastic strain. Some properties of die materials, such as the thermal expansion coefficient, thermal conductivity, yield strength, temper resistance, creep strength, and ductility, influence thermal fatigue. The thermal expansion coefficient should be as small as possible. High thermal conductivity reduces the thermal gradients and thermal stresses. A high heat yield strength lowers the plastic strain and improves resistance to thermal fatigue. The ratio of the tensile strength to the yield strength of 1.2709 steel is equal to 1.06. A significant decrease in material strength can only take place at temperatures above 500 °C. This means that the heat-checking damage accelerates slower. At present, there is no clearly-defined method for assessing the wear resistance of materials intended for creating parts of pressure mold tooling, especially those with surfaces that come into contact with liquid alloys. Under industrial conditions, the number of injections of molten metal into a mold is regarded as the wear limit; after reaching this limit, the casting either does not maintain its previously assumed dimensional tolerances or clearly-outlined mold surface cracks appear on its surface, which cannot be removed with the use of machining for economic or technological reasons. Several authors and publications [31] have presented a synthesized approach that addresses some of the problems related to the thermal fatigue of pressure mold inserts. In terms of the non-destructive testing group, using an ultrasound method to determine material fatigue under industrial conditions constitutes the interesting solution that is presented in [32]. The principles of carrying out a fatigue test adapted to determine the durability of those materials from which pressure mold inserts are made are provided in [33]. The service durability of pressure molds not only depends on the material from which they are made, but also on the applied thermal or thermo-chemical treatment that is used to increase the surface durability. Taking into consideration the difficulty of clearly defining the degree of wear that concerns mold parts made of maraging steel powders with the SLM process, we took advantage of an industrial research approach. For this purpose, a test sprue spreader with a conformal cooling channel system was created and installed in a pressure form.

## 2. Materials and Methods

Maraging steel in the form of CL50WS gas-atomized powder produced by AP&C-a GE Additive company (Québec, Canada) was used as the raw material. The chemical composition of the powder corresponds to steel marked with the 1.2709, DIN X3NiCoMoTi18-9-5, 18Ni(300) symbol, which is a high-strength, martensitic, and age-hardenable hot-work steel. The chemical composition of the powder (determined on the basis of spectroscopic tests) is listed in Table 2.

**Figure 1:**
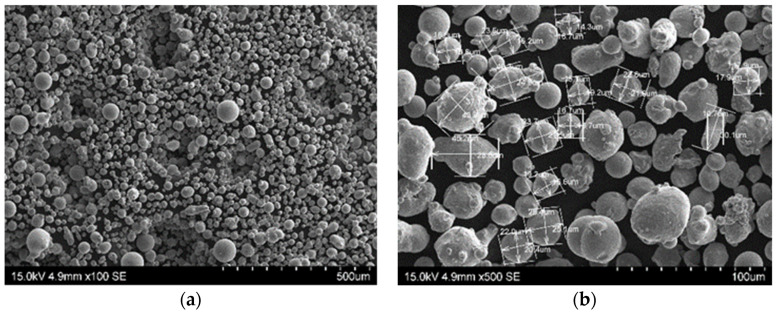
Scanning electron microscopy (SEM) morphology of 1.2709-grade maraging steel powder: (**a**) Image of steel powder; (**b**) different grain sizes marked.

**Figure 2:**
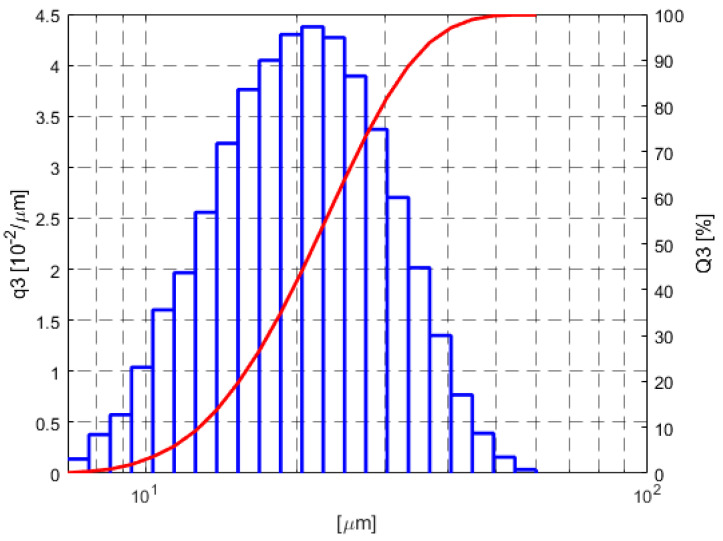
Size distribution of 1.2709-grade maraging steel powder.

Figure 1 shows the SEM morphology of the powder and Figure 2 shows its size distribution as measured by a Fritsch Analysette 22 NanoTech laser scattering particle size analyzer. The powder that was previously used to print SLM parts was used in the research. The powder particles had a median particle size of cumulative volume distribution x_50,3_ = 24.28 µm. The minimum and maximum particle sizes were x_min_ = 8 µm and x_max_ = 60 μm, respectively, and particles were spherical or quasi-spherical in shape (Figure 1). This shape allowed the particles to flow without hindrance during deposition. The samples for the testing and the sprue spreader were made using the M2 Concept Laser Cusing device. The material was printed in a nitrogen atmosphere with parameters as in Table 3.

The island scanning pattern was used in order to minimize the effect of thermal-induced stresses in the components. In this scanning strategy, part of the surface of each slice was divided into small square islands of 5 mm × 5 mm. The islands were scanned in a random manner, while the scanning direction was altered at a right angle with respect to the neighboring islands. This pattern is visible on the surface of the sprue spreader (Figure 12).

The relative density, *ρ*_r_, of the fabricated specimens was measured by formula *ρ*_r_ = (m_0_*ρ*_1_)/(m_0_*ρ*_0_-m_1_*ρ*_0_) according to Archimedes’ principle in which m_0_, *ρ*_0_, m_1_, and *ρ*_1_ are the maraging steel specimen’s weight in air, its theoretical full-density (8.05 g/cm^3^), its weight when submerged in water, and the density of the applied water under normal atmospheric pressure, respectively. The relative density of the fabricated specimens reached 99.12%.

Strength tests were carried out on samples with a measured length of 28 mm and a diameter of 5 mm cut from SLM-printed cylinders with a diameter of 35 mm and a height of 150 mm. The axis of each cylinder and the cut-out samples were perpendicular to the plane of the base plate of the SLM device. Such a position of the printed part in relation to the initial plate is usually least favorable in terms of the strength properties of the obtained material. In the case of the maraging steel, the difference in strength of the printed samples at angles of 45° and 90° in relation to the plate is not high, while greater differences occur in terms of the elongation values [34]. An MTS extensometer with a 20 mm base was used to measure elongation. A static tensile test was carried out at ambient temperature, and we tested eight samples for each variation of heat treatment and raw alloy. Metallographic tests of the microsections and fractures were performed using a Nikon SMZ 745T stereoscopic optical microscope (Tokyo, Japan), Nikon Eclipse metallographic microscope with a DsFi1 camera that enabled digital image analysis, and a HITACHI (Tokyo, Japan) S-3400N scanning electron microscope with Thermo Noran energy dispersive X-ray spectroscopy. Chemical composition tests were also carried out using energy dispersive X-ray fluorescence (ED-XRF) via a SPECTRO MIDEX spectrometer (Kleve, Germany). The heat treatment of the cylinders from which the samples were cut and made using the SLM printing method consisted of aging for one and eight hours at four different temperatures: 460, 540, 580, and 600 °C. The heating and cooling took place at a speed of 100 °C/h.

Based on the static tensile test results for the materials, specific heat treatment parameters were selected that gave the alloy an optimal ratio of strength and hardness to plasticity. Numerical calculations to determine the temperature field and stress field in the sprue spreader were performed with a Dassault System Simulia Abaqus v.2019. The coefficients describing the constitutive model of the cast alloy (Table 4) and maraging steel (Table 5) adopted in the numerical calculations were determined based on the database of the MAGMASOFT^®^ v5.4.1 and ProCAST v14.5 software, the EOS and Concept Laser datasheets, and the results of the experimental research.

## 3. Results

### 3.1. Microstructural Characterization of Built Samples

**Figure 3:**
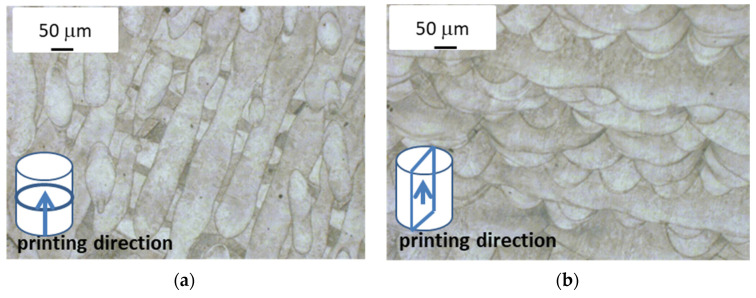
Images of printed alloy microstructure (optical microscope). (**a**) View direction perpendicular to direction of laser scanning path (top view); (**b**) view direction parallel to direction of laser scanning path (lateral view).

The images of the printed alloy structure in the raw state that were made by using optical microscopy revealed a characteristic feature of the SLM process, namely, the presence of interconnected or overlapping laser beam scanning paths. Depending on the location of the microsection’s plane in relation to the printing direction, the structure images differ, as shown in Figure 3.

**Figure 4:**
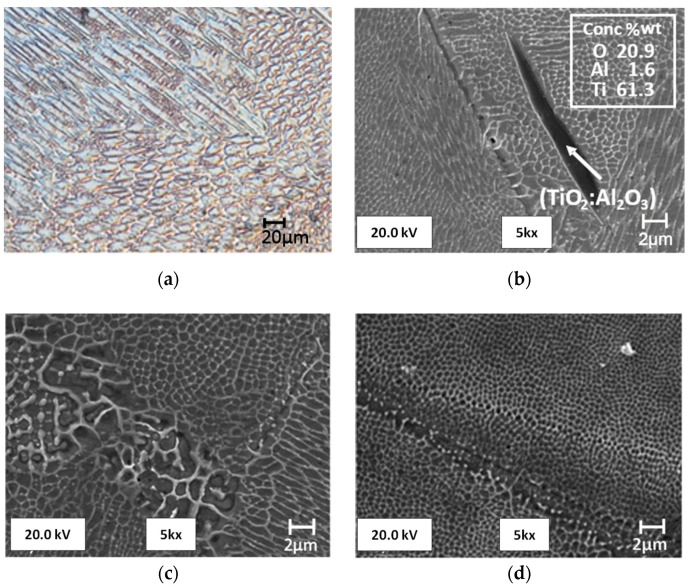
Image of alloy microstructure after solidification: (**a**) Elongated grains from the side view using an optical microscope; (**b**) oblong precipitate containing over 60%Ti present in alloy structure, taken with a scanning microscope; (**c**) different cell sizes in alloy structure, taken with a scanning microscope; (**d**) epitaxial growth of crystal lattice between scanning paths, taken with a scanning microscope.

The top view shown in Figure 3a reveals the presence of scanning paths in the form of wavy and, in some places, parallel lines. It is possible to observe semicircular cross-sections of scanning paths that overlap in Figure 3b that form the characteristic fish scale pattern. This semi-circular form results from the formation of a melting pool at the place where the powder and part of the underlying substrate is melted with the laser beam, which then solidifies into this characteristic form. The overlapping scanning paths that are visible in Figure 3b were intended to prevent the formation of any possible alloy discontinuity in the area between them, whereas changes in the direction of the scanning paths in adjacent layers (also visible in Figure 3b) were intended to reduce the natural stress that was generated in the alloy during printing. At a magnification of approximately 500×, it is possible to see the alloy structure after solidification that consists of elongated grains; this usually forms during the very fast heat dissipation that occurs during the SLM process as the melting pool solidifies (Figure 4a).

The amount of molten metal in the melting pool is small because its diameter is comparable to the diameter of the laser spot. An extremely large difference between the volume of the molten metal and the surrounding powder or solidified alloy works in favor of good heat conduction. The columnar grain growth depends on the temperature gradient and at high values helps to form a fine-grained structure. This microstructure is significantly different from a typical martensite microstructure, which contains primary austenite grains as well as martensite packages, blocks, and tiles. However, the presence of residual austenite and martensite in the structure was confirmed with the use of the electron back-scattered diffraction (EBSD) method [35]. In the images from a scanning microscope, the alloy structure formed during crystallization is composed of various cell sizes (Figure 4c) in which there are often precipitates that contain mainly Ti. The presence of Ti inclusions or oxides (TiO_2_:Al_2_O_3_) with a size of 10–20 μm was also confirmed by [23], and it worsens the properties of the alloy, especially after aging, making it brittle.

The SEM-EDS analysis carried out on the samples confirmed the presence of this type of precipitation (Figure 4b). Figure 4b,d shows epitaxial cell growth, which duplicates the existing crystal lattice arrangement between two scanning paths. This phenomenon is characteristic of the microstructure of an alloy obtained by the SLM method, which was confirmed in the literature [16,36,37]. The material obtained when using the SLM method may include defects that reduce its mechanical properties. Their numbers and size depend on the adopted operating parameters of the SLM device.

### 3.2. Defect Analysis

**Figure 5:**
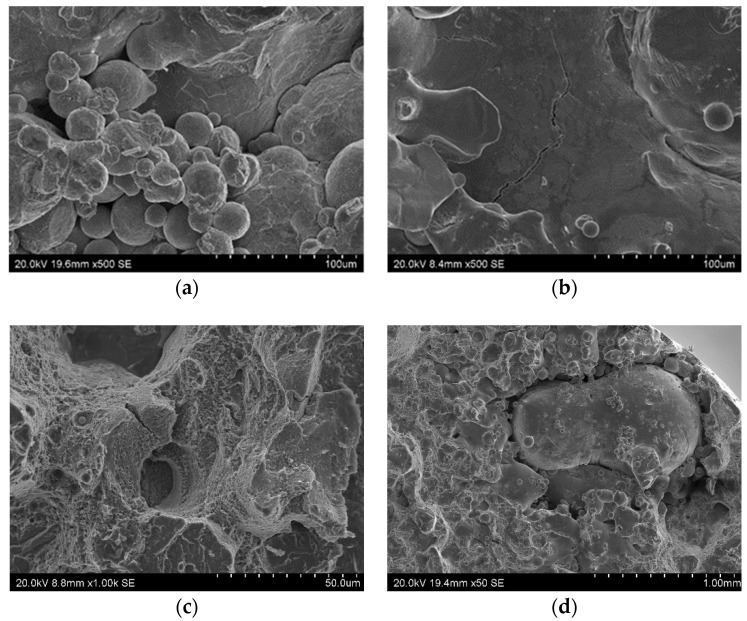
Defects found in sample fractures (scanning microscope): (**a**) A large cluster of partially-melted powder (alloy after aging); (**b**) a crack in the zone containing powder blown out by argon (alloy in raw state); (**c**) two craters connected by a crack (alloy after aging); (**d**) a large defect caused by the incorrect fusion of powder (alloy in raw state).

**Figure 6:**
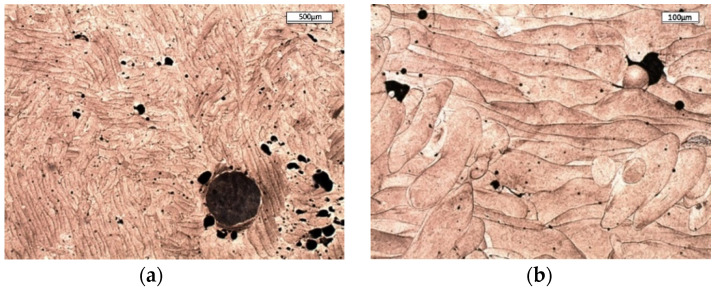
Porosity in printed alloy (as-built material), taken with an optical microscope at (**a**) low; and (**b**) high magnifications.

Defects are visible both on the metallographic microstructures and in the scanning images of the fracture surfaces of the samples made after the static tensile test. Among the most common defects that occur in the alloy structures are large clusters of partially-molten powder (Figure 5a), cracks (Figure 5b), craters with cracks appearing next to them (Figure 5c), and defects caused by incorrect powder melting (Figure 5d), while the most common defect is alloy porosity, which is shown in Figure 6.

**Figure 7:**
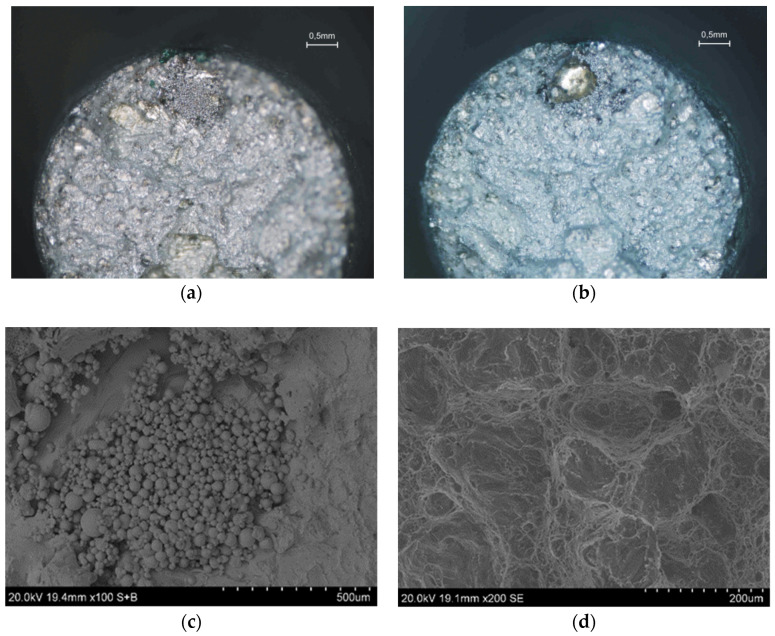
Macroscopic image of sample fractures with unmerged powder area (**a**,**b**); (**c**) unmerged powder area; (**d**) sample fracture outside unmerged powder area.

Figure 7a,b shows two parts of the same sample surface fractures as a result of a static tensile test. The defect, which significantly reduces the sample’s strength, consists of unmerged spherical powder (shown in Figure 7a,c), which corresponds to the solid melted structure of the sample from Figure 7b. The presence of unmelted material was occasionally observed in the samples, as indicated by results of the chemical composition tests carried out in selected areas of the sample made with the ED-XRF method. This fact does not result from the material’s heterogeneous chemical composition, but is rather an effect of the printing process itself.

**Figure 8:**
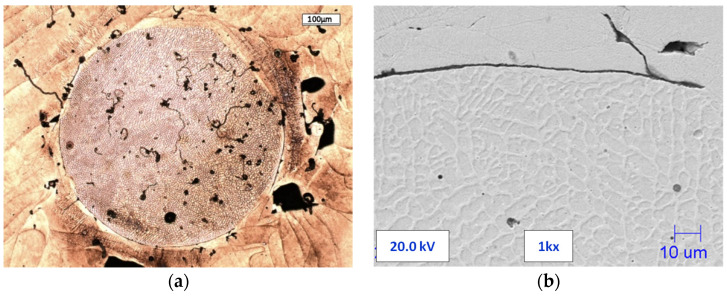
Processed particles: (**a**) Optical microscope image of an entire particle with porosity and cracks; (**b**) SEM image of fracture at the boundary of a particle and material.

The reason for the formation of a crack in the printed material may be the individual particles that were created as a result of blowing out the metal by argon from the melting zone. Their diameters are usually several times larger than those of other powder particles. An example of such a crack in the vicinity of a particle with a diameter of circa 550 μm is shown in Figure 8.

### 3.3. Tensile Test and Fracture Surface Analysis

**Figure 9:**
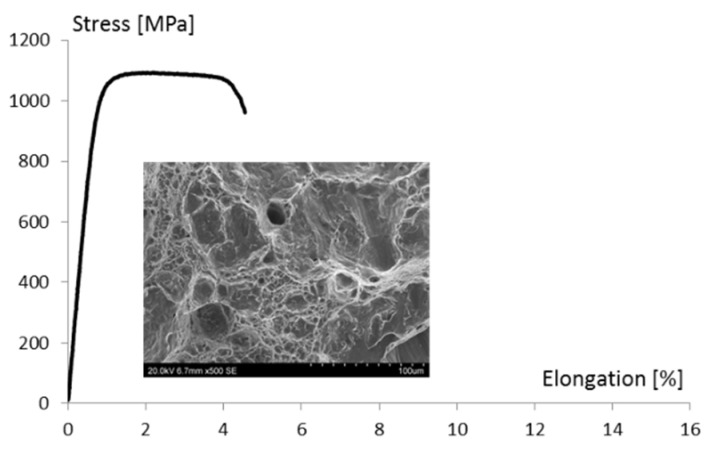
Typical tensile curves obtained for tested raw alloy and images of sample fractures.

The printed alloy tensile tests were carried out in the raw state and after heat treatment. Figure 9 shows a typical elongation graph, in which the stress was obtained during the tensile tests of the raw material. These charts include a characteristic plateau (between 1 and 4% of the elongation) without a clear location of the maximum force. The ultimate tensile strength (UTS) of the raw printed alloy exceeded 1100 MPa, while Elongation A was about 4%. The sample’s fracture shows the presence of yield deformation zones (Figure 8).

**Figure 10:**
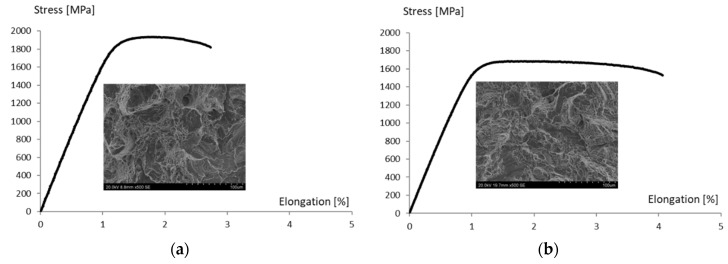
Typical tensile curves obtained for tested alloy after heat treatment and images of sample fractures: (**a**) Temperature of 540 °C and duration of 1 h; (**b**) temperature of 540 °C and duration of 8 h.

Figure 10 shows typical graphs of the elongation function, with the stress obtained during the tensile tests of the material after aging at 540 °C for one and eight hours. An increase in the aging temperature to above 540 °C results in a decrease in tensile strength UTS and a conventional yield strength of R_p0.2_, while simultaneously increasing the alloy’s yield and elongation (Table 6). Reducing the duration of the heat treatment increases the strength and decreases the elongation of the material. In Figure 10a, there is an increase in the share of the brittle fracture compared to the fracture in Figure 10b.

### 3.4. Sprue Spreader with Conformal Cooling System

#### 3.4.1. Design of Cooling Channels

**Figure 11:**
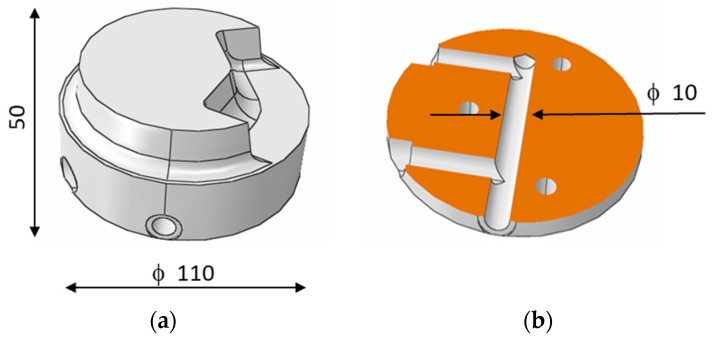
Computer Aided Design (CAD) drawing of sprue spreader: (**a**) Outside view, diameter of spreader’s outer profile is 110 mm and its height is 50 mm; (**b**) conformal channel design, diameter of 10 mm.

The Computer Aided Design (CAD) model of a spreader with a classic cooling system solution is presented in Figure 11. The diameter of the spreader’s outer profile was 110 mm and its height was 50 mm (Figure 11a) and the cooling channels had a diameter of 10 mm. The length of each of the two shorter channels was 45 mm, while the length of the third cooling channel was 90 mm (Figure 11b)**.**

**Figure 12:**
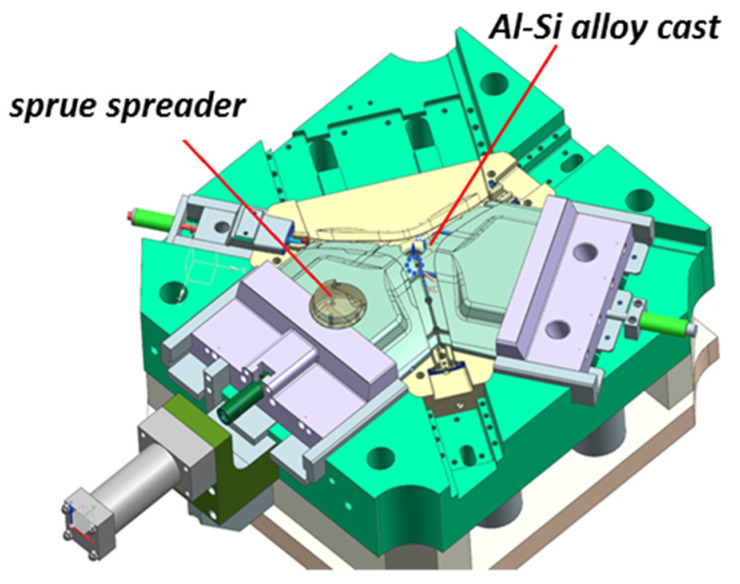
Sprue spreader used in die casting of the AlSi alloy.

In the classic version, the spreader was made of H13 steel. The cooling channels were drilled using the CNC method. The location of the spreader and the casting in the pressure mold are shown in Figure 12.

**Figure 13:**
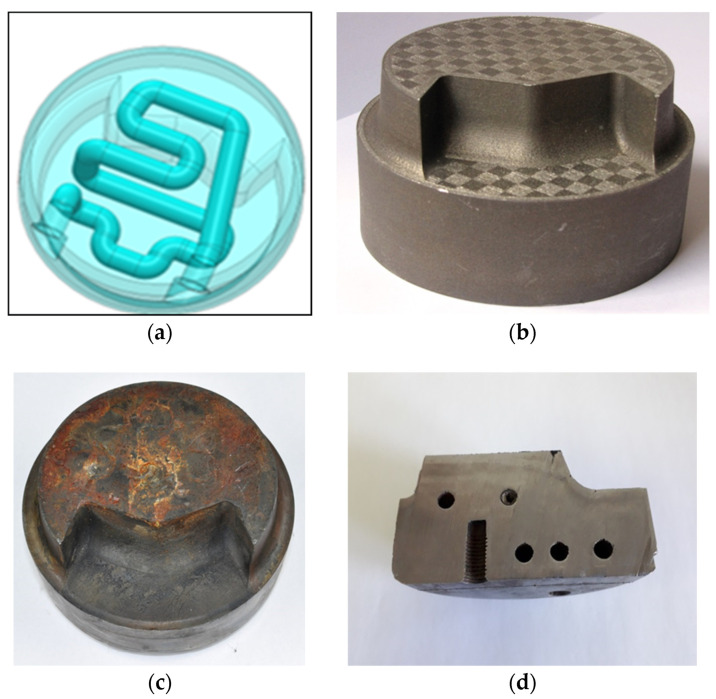
Sprue spreader with conformal cooling channels: (**a**) CAD drawing of conformal cooling channels; (**b**) image of sprue spreader manufactured by additive SLM method; (**c**) tested sprue spreader after tens of thousands of operating cycles; (**d**) tested sprue spreader with visible arrangement of conformal channels.

In order to increase the cooling efficiency, a conformal channel system was designed (Figure 13a). The external form of the parts did not change, that is, they were the same as in the case of the spreader with a classic cooling system. Figure 13b presents a spreader printed with the SLM method. Figure 13c,d shows the same element removed from the pressure machine after about 40,000 operating cycles in order to check the surface condition and the potency of the channels.

#### 3.4.2. Heat Flow Model

**Figure 14:**
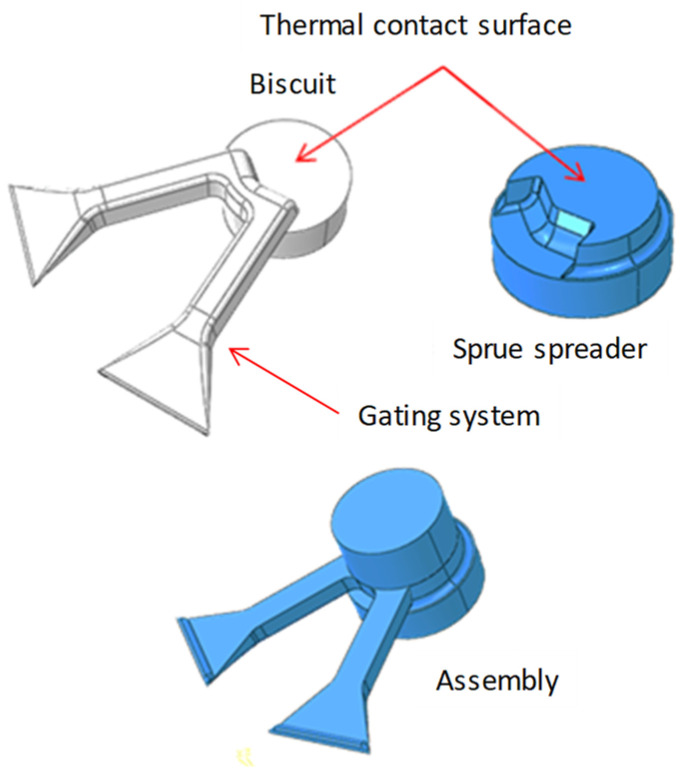
Geometrical model of thermal contact surface between sprue spreader and biscuit.

FEM numerical models were implemented to determine the temperature changes in spreaders with classic and conformal cooling systems during the operation of a pressure machine based on CAD drawings (Figure 14).

The FEM calculations used temperature-dependent data for conductivity, specific heat, latent heat (AlSi alloy), and density. The databases of the MAGMASOFT^®^ and ProCAST software were used. The thermal contact on the thermal contact surface and the thermal conductance on other surfaces are defined in the Dassault System Simulia Abaqus. These coefficients were determined on the basis of the MAGMASOFT^®^ and ProCAST databases. It was assumed that the temperature in the channel axis was constant. The temperature fields are shown after 20 work cycles. The contact effects were modeled using surface-based interaction definitions. The surfaces of the mold and alloy were physically in contact with each other but have distinct temperatures on their respective sides. Convective surface conditions occur between the channel surface and the cooling liquid, as defined by the following equation:

(1) $$q=-\alpha_{k}\left(T_{C}-T_{L}\right),$$

where *q* is the heat flux, *T_C_* is the surface temperature of the cooling channel, *T_L_* is the temperature of fluid adjacent to surface *C*, and *α_k_* is the film coefficient.

The film coefficient was determined based on the Schack formula [38]:

(2) $$\alpha_{k}=3370\cdot \left(1+0.014t_{m}\right)\cdot {W_{m}}^{0.85},$$

where *W_m_* is the water velocity, and *t_m_* = 35 °C is the average water temperature in channel.

In the case when the rectilinear sections of the channel with diameter *d_k_* are short and there are many curves, the correction for the radius of curvature r_k_ was taken into account:

(3) $${\alpha^{\prime }}_{k}=\alpha_{k}\left(1+1.77\frac{d_{k}}{r_{k}}\right).$$

**Figure 15:**
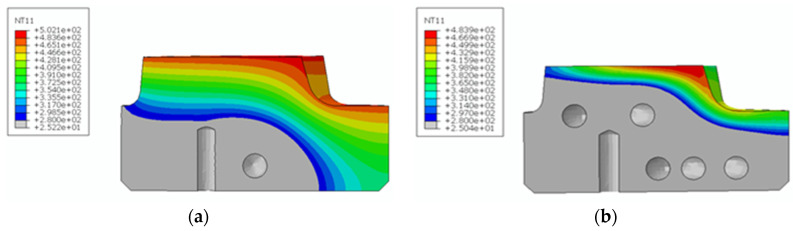
Location of the 280 °C isotherm in the sprue spreader: (**a**) Usual solution of cooling channels; (**b**) conformal cooling channels. NT11—temperature in °C.

The working cycle of the pressure machine consists of the following phases: Mold closure, alloy injection, alloy solidification and cooling, mold opening, casting removal, spraying the surface of the mold with separating liquid, and re-closure. The boundary conditions on the upper surface of the sprue spreader change during the injection, solidification, and cooling phases of the pressure machine operational cycle, whereby a thermal contact of the alloy and the surface of the sprue spreader occurs. After opening the mold and removing the casting, convection occurs between the mold surface and the air. During the phase of spraying the mold with separating liquid, there is a much more intense phenomenon of convection between the upper surface of the sprue spreader and the liquid. The calculations of the temperature changes during the successive phases are repeated until the time (the number of working cycles) when the temperature on the other surfaces of the sprue spreader that were not in contact with the alloy reaches the average mold temperature. The casting injection and solidification phase in the next cycle of the pressure machine operation is representative of the stable temperature conditions of the mold and, thus, the machine’s operation. After about 20 cycles, the temperature stabilizes in the mold. Here, we present the temperature field and stress field results when the mold has already reached a stable operating temperature. The calculations were carried out assuming the time from injecting the metal into the mold to removing the casting from the mold (τ_push_) is 7 s and the initial melt temperature T_init_ = 680 °C. For castings with an average wall thickness from 3 to 6 mm, the zone of the temperature field changes in the upper layers of the mold cavity during the production cycles does not exceed a depth of 5–6 mm [38,39]. As the thickness of the casting wall increases, the depth of this zone and the average mold temperature increases. The spreader example constitutes a certain marginal case for the magnitude of these changes due to the large wall thickness of the sprue biscuit of about 30 mm. In the case of such thick walls, the average temperature of a spreader with a classic arrangement of cooling channels is 280 °C according to FEM calculations. Figure 15 shows the location of this isotherm in the analyzed variants of spreader cooling systems.

**Figure 16:**
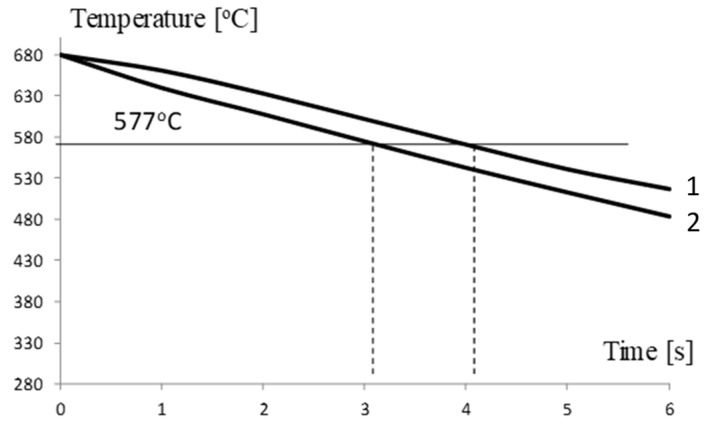
Temperature changes on sprue spreader contact surface: Curve 1—traditional solution; Curve 2—conformal cooling channel system.

Our calculations reveal that the 280 °C isotherm in a spreader with a classic cooling system is located approximately 25 mm from the contact surface of the spreader and the cast alloy (Figure 15a). The location of the 280 °C isotherm changes using a conformal channel system. Its average distance from the top surface of the sprue spreader decreases to 9 mm (Figure 15b). In the area bounded by the 280 °C isotherm and the upper surface of the spreader, there are cyclical changes in the temperature-stress field during successive operation cycles of the pressure machine. Using a conformal channel system leads to a reduction in the solidification time of the metal in the sprue biscuit. The time taken for the temperature to drop to 577 °C at the contact surface between the spreader and alloy is 1 s shorter in the case of a conformal cooling system when compared to a traditional cooling channel system (Figure 16).

#### 3.4.3. Stress State Model

The components of the stress tensor in the sprue spreader model were determined based on the calculated changes in the temperature values in the heat flow model. The applied temperature-dependent data for thermal expansion, Young’s modulus, and the yield stress from the databases of the MAGMASOFT^®^ and ProCAST software were used. The calculations took the pressure exerted by the AlSi alloy on the sprue spreader surface into account. The sequential stress procedure was used in the calculations. In this case, the temperature solution depends on the position and time and is read into the stress analysis as a predefined field. Thermal strains, ε, at the element material locations are calculated according to the following equation:

(4) $$\varepsilon =\alpha \left(\theta \right)\left(\theta -\theta^{0}\right)-\alpha \left(\theta^{INT}\right)\left(\theta^{INT}-\theta^{0}\right),$$

where *θ* is the current temperature, *θ^INT^* is the initial temperature, *θ^0^* is the reference temperature for the expansion coefficient, and *α(θ)* is the coefficient of thermal expansion.

**Figure 17:**
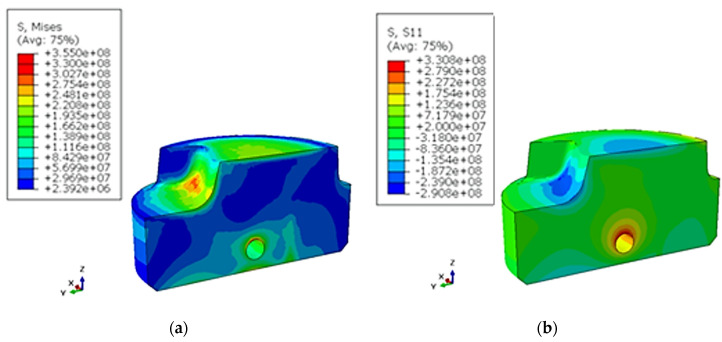
Image of thermal stress [Pa] caused by temperature field changes calculated with FEM directly after injection in a traditional channel system: (**a**) σ_o_ according to Huber–Mises HM hypothesis; (**b**) stress components σ_x_.

**Figure 18:**
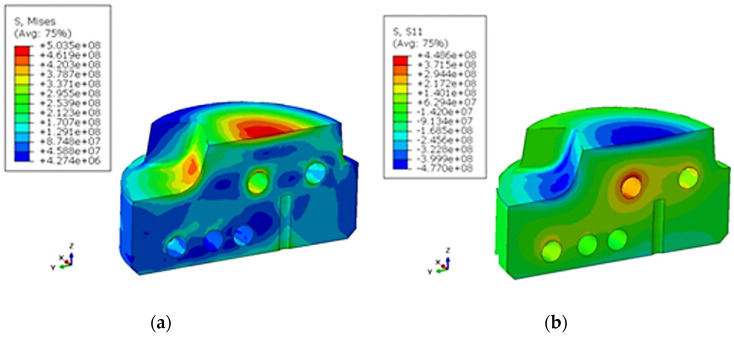
Image of thermal stress (Pa) caused by temperature field changes calculated by FEM directly after injection in a conformal channel system: (**a**) σ_o_ according to HM hypothesis; (**b**) stress components σ_x_.

Changing the design of the channels significantly increased the surface areas that were cooled by liquid. Therefore, a more intense heat exchange occurs, which has an impact on reducing the average operating temperature of the spreader. A visible result of the more efficient operation of the cooling channels can be obtained by shifting the location of the 280 °C isotherm towards the contact surface with the alloy. A phenomenon that can limit the efficiency increase of a conformal mold cooling system consists of generating too much stress by such solutions. The reason for this is the intensive temperature changes that cause thermal stress, which depends on the temporal and spatial temperature gradient as well as the coefficient of the thermal expansion concerning the material from which the mold cooling system element was made. Figure 17 and Figure 18 shown the reduced stress field σ_o_ calculated using FEM according to the Huber–Mises (HM) hypothesis and stress state component σ_x_ for conventional and conformal cooling systems.

## 4. Discussion

**Figure 19:**
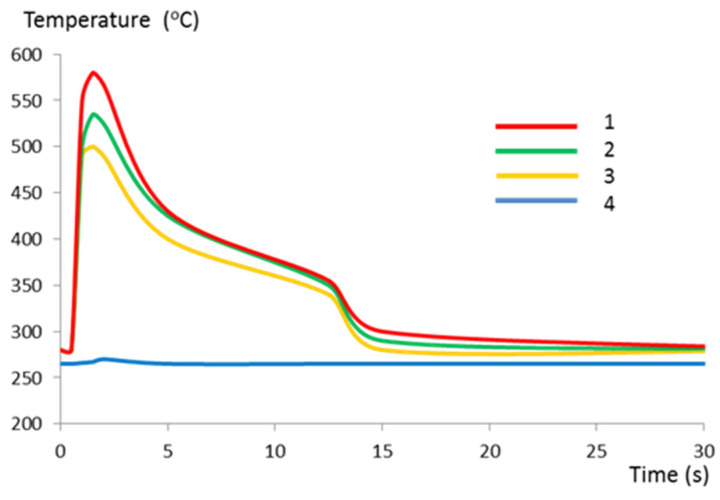
Temperature changes during one cycle of pressure machine operation in a sprue spreader with a conformal cooling system at different distances from the contact surface to the cast alloy: Curve 1, 0.5 mm; curve 2, 3.0 mm; curve 3, 5.0 mm; curve 4, 9.0 mm.

Pressure mold inserts, cores, and gates are typically machined from H13 steel, which has a good combination of high strength and hardness with plastic properties. Due to its high content of carbon (C), which is ten times greater than that of maraging 1.2709, it is of limited use in the SLM method. In the process of making parts of the pressure mold tooling from maraging steel powders, it is important to have good part designs, which take into account the specificity of SLM technology; to ensure the proper performance of the SLM process; and to select the appropriate heat treatment of the mold. Due to the efficiency of the uniform heat transfer from the metal, the cooling channels made using the SLM method should be located close to the mold surface. On the other hand, reducing the distance between the channel surface and the mold surface increases the stresses. The recommended average mold temperature for die-casting aluminum alloys is 230–280 °C. This temperature is constant during the press cycles of the pressure machine in almost the entire volume of the mold, except for the area near the surface of the mold. In this zone, the temperature value is directly influenced by changes in the boundary conditions that occur during the injection of metal at a temperature of 680 °C, the solidification and cooling of the alloy, opening of the mold halves, and pouring out the casting and spraying with an oil-water mixture at a temperature of about 30 °C, which causes the temperature of the sprue spreader surface to drop by about 100 °C. Figure 19 shows the changes in temperature during one cycle of pressure machine operation in the section plane passing through the center of the sprue spreader with a conformal cooling system at different distances from the contact surface to the cast alloy.

In a sprue spreader with a conventional cooling system, the zone of the mean temperature constant over time, corresponding to curve 4 in Figure 19, occurs at a distance of about 25 mm from the contact surface to the cast alloy. In the case of using conformal channels, the zone of stable temperature is already approximately 10 mm from the upper surface of the sprue spreader. The difference between the position of the 280 °C isotherm in both sprue spreaders results from the reduction of the distance between the surface of the conformal cooling channels and the surface of the mold cavity by about 25 mm (Figure 15b) and the greater length and cooling surface area of these channels compared to the conventional channel system (Figure 15a). The cooling surface of conformal channels (11,500 mm^2^) is twice as large as the distributor with the classic arrangement of cooling channels (5652 mm^2^). According to theoretical calculations based on the heat balance used for some of the pressure molds, this area should be at least 7562 mm^2^ (Table 7), but the use of CNC technology to drill straight-line channels did not provide them with an adequate heat exchange surface.

We analyzed the cooling system of the sprue spreader, that is, the part of the mold that has no direct impact on the quality of the casting. The conformal arrangement of the channels shortens the time of solidification of the biscuit compared to the classic solution of the cooling system. Temperature changes (Figure 16) on the contact surface with the metal of both sprue spreaders show that the temperature of 577 °C (eutectic solidification point) will occur one second earlier for the conformal cooling system. The authors of [1], who used a conformal cooling system in a pressure mold for casting Zn alloys, also reported a reduction in the solidification phase time by 1 s. If a conformal cooling system is used in the pressure mold insert, the process of metal injection into the mold and the solidification of the alloy can be controlled. Taking into account that excessive thermal stresses caused by excessive temperature gradients should not arise in the mold leads to the establishment of estimated minimum distances between the channel and the cavity. This distance for Al alloys should not be less than 15 mm. In molds with a conformal cooling system, this distance is usually much smaller. In some designs of pressure molds, the distance between the channels and the mold cavity surface is reduced to 2.5 mm [1]. The same study also reported that the failure-free number of cycles of the mold was 2000 cycles. For this reason, testing the mechanical and strength properties of the material produced by the SLM method is of great importance.

CL 50WS powder was used to produce the spreader, with the powder’s chemical composition corresponding to 1.2709-grade maraging steel. This powder was used previously to print other parts. The samples and the sprue spreader itself were printed while maintaining the same SLM device parameters. The resulting material had an apparent density of 99.12% (slightly lower than the assumed 99.90%). This resulted from the adopted printing parameters, including a rather high production speed [40]. Defects in the form of un-melted metal powder aggregates, cracks, porosity, and precipitates with a high Ti content were found in the obtained material; these defects reduced the materials’ strength properties.

The microstructure of the printed material contains spherically shaped un-melted particles sometimes, which are much larger than powder particles. They are formed as a result of the gas blowing a portion of the metal from the melting pool. During the SLM process, these particles are not remelted and fuse with the rest of the material due to their large diameter. They may act as starting points for defects and crack initiation, as they are thicker than the material layer. Photos presenting the morphology of the powder include visible powder particles with a size of about 70 µm, and their presence was also confirmed by tests of the size distribution of the particle’s powder. The dimensions of these particles are larger than the layer thickness of 50 μm. In addition, there were some particles with diameters that were several times larger than the thickness of the layer (Figure 8).

The presence of defects had the greatest impact on reducing the value of the elongation recorded during the tensile tests. Based on a static tensile test, a heat treatment variant (alloy aging at 540 ℃ for 1 h) was selected, which gave the material a high strength and relatively good plasticity. The heat treatment recommended by the powder manufacturer consists of aging for 6 to 10 h at 540 ℃. The material aged at 540 ℃ for 8 h showed an insufficient hardness (about 47 HRC) during the tests when compared to a hardness of above 50 HRC that the surface of a spreader that is in direct contact with a liquid alloy should possess. The mechanical properties of the material aged for 8 h are more suitable for use in producing pressure mold inserts. A stress analysis based on FEM calculations clearly indicates that, regardless of the cooling system, compressive stresses are dominant in the surface layers of spreaders. The calculated values of the compressive stresses for a classic cooling system do not exceed 330 MPa; this number is 450 MPa for a conformal system. The above-mentioned stress values occur directly on the surface of contact with solidifying metal. A stress analysis in deeper layers indicates an incremental decrease in their values. At a distance of 0.5 mm from the contact surface to the metal, the stress decreases to 135 and 158 MPa for conventional and conformal cooling systems, respectively. A triaxial state of stress occurs in the spreader body. Therefore, an evaluation of the material strength has been carried out on the basis of an HM hypothesis. The reduced stresses on the surface of a metal’s contact with a spreader’s surface are 355 and 503 MPa for conventional and conformal cooling systems, respectively. The stress values in spreaders determined with FEM were lower than the conventional yield strength R_p0.2_ of a material obtained with the SLM method for both cooling channel systems. After opening the mold, removing the casting, and applying a lubricant, the stresses changed to a positive marking on the surface of the cavity. The value of the tensile stress was much lower than the compressive stress. However, a fatigue cycle was created, which resulted in the initiation of fractures on the surface of the mold cavity. Due to this, the final verification of the pressure mold structure consisted of time-consuming industrial tests to determine the relationship between the number of cycles and the condition of a mold surface.

## 5. Conclusions

The tests we conducted confirm the possibility of using SLM technology to implement parts of a pressure mold cooling system, thanks to both the high-strength properties of the obtained printed material and the features of this technology. The SLM method makes it possible to implement parts of the mold that are optimal in terms of heat dissipation from the solidifying alloy and whose construction is non-technological when using CNC machining methods. With the use of a numerical model of the thermo-mechanical loads of a mold’s surface layers, an analysis of the operation of conventional and conformal cooling channel systems has been carried out in terms of their efficiency in transferring heat from a casting. It has been demonstrated that using a conformal channel system intensifies the heat dissipation from a solidifying alloy and reduces the time from the moment of metal injection to removing the casting from the mold by about 1 s. The use of a conformal cooling system lowers the average temperature of the sprue spreader in the cooling channel zone by about 20 °C. However, the related increase in the temperature gradient concerning a mold’s surface layers does not cause such a large increase in the value of the thermal stress that it significantly reduces the durability of this part of the mold. We based these conclusions on observations of the technical conditions of spreaders removed from molds after certain periods of operation. Due to the time-consuming nature of conducting tests under industrial production, the generalization of such results is limited. The tests conducted on the material showed its good mechanical properties; however, these can be significantly deteriorated through the defects that arise when using the SLM method. Shortening the aging time and lowering the temperature to within a range of 450–600 °C increases the hardness of the surface and the strength of the material while reducing its elongation. Differences in the share of plastic and fragile fractures were also observed in the SEM images of the sample fractures depending on the applied heat treatment. Taking the function served by a spreader in a pressure mold into consideration, such heat treatment parameters were adopted for the material from which it is made, which provides a surface hardness of over 50 HRC. The proposed spreader construction solution has been verified during operation under industrial conditions.

## Figures and Tables

**Table 1:** Chemical composition wt% of CL 50WS, MS1, and Marlok C 1650 powders as well as H13 steel.

Steel or Powder Grade	C	Si	Mn	P	S	Cr	Mo	Ni	Ti	Co	Al	V
H13 [16]	<0.36	1.0	0.44	-	-	5.3	1.3	-	-	-	-	0.98
Marlok C1650 [17]	<0.01	<0.1	<0.01	<0.01	<0.005	<0.2	3–6	13–16	<0.3	10–13	-	-
CL 50WS [18]	<0.03	<0.1	<0.15	<0.01	<0.01	<0.25	4.5–5.2	17–19	0.8–1.2	8.5–10.0	-	-
MS1 [19]	<0.03	<0.1	<0.10	<0.01	<0.01	<0.50	4.5–5.2	17–19	0.6–0.8	8.5–9.5	0.05–0.15	-

**Table 2:** Chemical composition wt% of studied powder.

Chemical Composition wt%				
**Ni**	**Mo**	**Co**	**Ti**	**Fe**
18.0	5.0	9.7	1.1	65.3

**Table 3:** Selective laser melting (SLM) process parameters.

SLM Parameter	Solid Section	Surface
Laser power (W)	340	180
Laser speed (mm/s)	1100	200
Laser beam diameter (µm)	120	50
Layer thickness (µm)	50	25
Hatch space (µm)	91	35

**Table 4:** The value of function describing the constituteive model of the cast alloy AlSi11.

Temperature	20 °C	550 °C	577 °C	600 °C	750 °C
Conductivity (W/m·°C) ^1^	160	177	112	80	80
Specific heat (J/kg·°C) ^2^	1060	1095	1095	1095	1095
Density (kg/m^3^) ^3^	2650	2600	2570	2490	2448
Latent heat (J/kg) ^4^	390	390	390	390	390

^1, 2, 3, 4^ database of MAGMASOFT^®^ and ProCAST software.

**Table 5:** The value of function describing the constitutive model of maraging steel (mold).

Temperature	20 °C	400 °C	650 °C
Conductivity (W/m·°C) ^1^	20	20	20
Specific heat (J/kg·°C) ^2^	450	450	450
Density (kg/m^3^) ^3^	8100	8100	8100
UTS (MPa) ^4^	1920	1900	960
R_p0.2_ (MPa) ^5^	1900	1864	930
Poisson’s ratio ^6^	0.27	0.27	0.27
Young’s modulus (MPa) ^7^	170,000	170,000	170,000
**Temperature Range**	**25–100 °C**	**25–200 °C**	**25–300 °C**
Linear expansion coefficient (1/°C) ^8^	10.7 × 10^−6^	11.2 × 10^−6^	11.5 × 10^−6^

^1, 2, 3, 6, 8^ EOS, Concept laser; ^4, 5, 7^ Experimental research.

**Table 6:** Tensile test results and hardness at different times and aging temperatures.

Aging	UTS (MPa)	R_p0.2_ (MPa)	A (%)	E (GPa)	HRC
As-built material	1117 (±26)	1057 (±31)	6.9 (±1.2)	145 (±7)	37
460 °C/8 h	2017 (±12)	1955 (±11)	1.2 (±0.1)	172 (±0.5)	57
540 °C/1 h	1920 (±14)	1864 (±37)	2.8 (±0.4)	168 (±2)	53
540 °C/8 h	1690 (±10)	1640 (±11)	3.8 (±0.2)	166 (±1)	47
580 °C/1 h	1600 (±21)	1529 (±20)	6.1 (±0.2)	165 (±1)	45
580 °C/8 h	1409 (±23)	1316 (±27)	9.4 (±0.8)	162 (±0.4)	42
600 °C/1 h	1418 (±35)	1357 (±30)	11.2 (±0.4)	162 (±0.2)	40
600 °C/8 h	1314 (±12)	1212 (±4)	12.4 (±0.2)	160 (±1)	39

**Table 7:** Comparison of channel lengths (L) determined on the basis of CAD drawings and the heat balance as well as the size of the cooling surface (F) for conformal and conventional solutions of pressure mold cooling system.

L_b_ (mm)	L_cl_ (mm)	L_co_ (mm)	F_b_ (mm^2^)	F_cl_ (mm^2^)	F_co_ (mm^2^)
240	180	366	7562	5652	11,500
Definitions: L_b_—cooling channel length calculated on basis of heat balance; F_b_—cooling channel area calculated on basis of heat balance; L_cl_—channel length in conventional cooling system based on CAD drawing; F_cl_—channel area in conventional cooling system based on CAD drawing; L_co_—channel length in conformal cooling system based on CAD drawing; F_co_—channel area in conformal cooling system based on CAD drawing.					
The following have been assumed in the calculations: Biscuit volume, V = 0.0348 m^3^; contact surface of spreader with metal F = 0.00032 m^2^; water flow speed, W = 2 m/s; channel surface temperature T_ch_ = 120 °C; water temperature T_w_ = 35 ℃, initial alloy temperature T_init_ = 680 ℃; casting push out temperature T_push_ = 400 ℃; alloy solidification heat q = 390 kJ/kg·℃; specific heat, c = 963 J/kg·℃; density ρ = 2700 kg/m^3^.					
